# Supplementary material for: RAC1B Induces SMAD7 via USP26 to Suppress TGFβ1-Dependent Cell Migration in Mesenchymal-Subtype Carcinoma Cells
Source: Cancers (Basel). 2020 Jun 11;12(6):1545. doi: 10.3390/cancers12061545 (PMC7352540; doi:10.3390/cancers12061545)
Supplement: Supplementary file 1 [file cancers-12-01545-s001.pdf]

Supplementary Materials

RAC1B Induces SMAD7 via USP26 to Suppress TGFβ1-Dependent Cell Migration in Mesenchymal-Subtype Carcinoma Cells

Hendrik Ungefroren, Anuradha Kumarasinghe, Melina Musfeldt, Christian Fiedler, Hendrik Lehnert and Jens-Uwe Marquardt

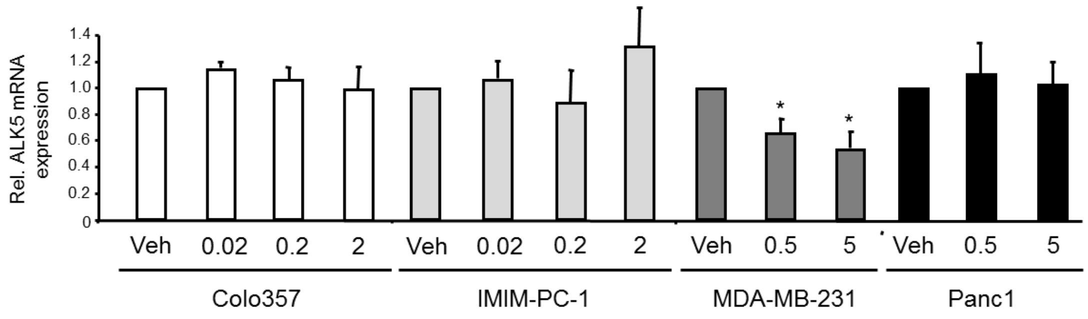

Figure S1. Effect of MG132 treatment on ALK5 mRNA abundance in various cell lines. Colo357, IMIMPC-1, MDA-MB-231 and Panc1 cells were treated for 24 h with the indicated concentrations of MG132, or vehicle (Veh). At the end of the incubation period cells were processed for qPCR analysis of ALK5, and TBP as internal control. Data represent the means ± SD of three parallel wells and are representative of three experiments. Data are plotted relative to the respective vehicle-treated control cells set arbitrarily to 1.0. Significant differences ($p < 0.05$, two-tailed unpaired Students' t test) relative to the corresponding vehicle-treated control are indicated by an asterisk.

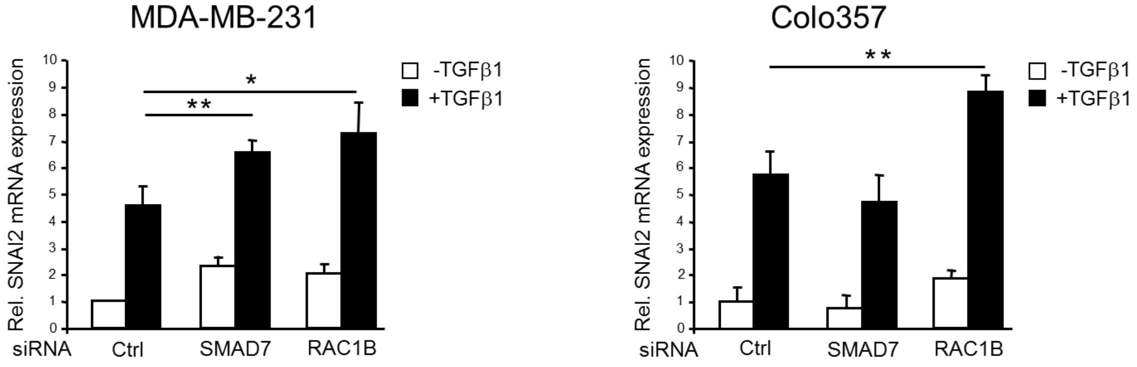

Figure S2. Effect of SMAD7 knockdown on SNAI2 expression in MDA-MB-231 and Colo357 cells. Both cell lines were transiently transfected twice on two consecutive days with 50 nM each of either control (Ctrl) siRNA, SMAD7 siRNA, or RAC1B siRNA (as positive control) and subsequently treated with TGF-β1 for 24 h. Total RNA was reverse transcribed and subjected to qPCR of SNAI2. Data represent the mean ± SD of three experiments.

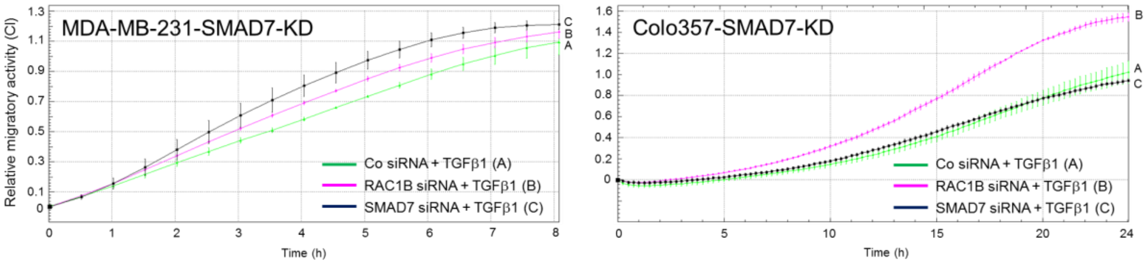

Figure S3. Effect of knock down of SMAD7 or RAC1B on TGFβ1-induced cell migration in MDA-MB-231 and Colo357 cells. Both cell lines were transfected twice on two consecutive days with 50 nM of irrelevant control (Co) siRNA, SMAD7 siRNA, or RAC1B siRNA as positive control. Forty-eight h later the transfected cells were subjected to real-time cell migration assay (chemokinesis setup) in the presence of 5 ng/mL TGFβ1. Data are the mean ± SD of 3–4 wells per condition and are representative of three experiments with very similar results. Differences between CosiRNA + TGFβ1 (green curve, tracing A) and SMAD7 si RNA + TGFβ1 (black curve, tracing C) are significant at 2:30 and all later time points, while differences between Co siRNA + TGFβ1 and RAC1B siRNA + TGFβ1 (magenta curve, tracing B) are significant between03:15and7:00.

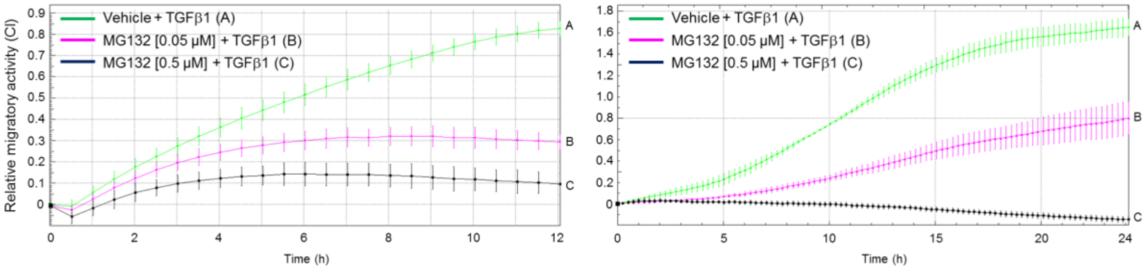

Figure S4. TGFβ-induced migration of RAC1B-deficient Panc1 cells and MDA-MB-231 cells is highly sensitive to proteasome inhibition. Panc1-RAC1B-KO or non-engineered MDA-MB-231 cells were subjected to real-time cell migration assay in the absence or presence of the indicated concentrations of MG132 and TGFβ1 (5 ng/mL). The assays shown are representative of 3 assays performed in total (mean ± SD of 3 wells). Left-hand graph: Differences between vehicle + TGFβ1-treated cells (green curve, tracing A) and MG132 + TGFβ1-treated cells are first significant at 03:00 (0.05 μM concentration, magenta curve, tracing B) and all later time points, or at 02:00 (0.5 μM concentration, black curve, tracing C).Right-hand graph: Differences between vehicle+ TGFβ1-treated cells (green curve, tracing A) and MG132 + TGFβ1-treated cells are first significant at 03:00 (0.05 μM concentration, magenta curve, tracing B) and all later time points, or at 03:00 (0.5 μM concentration, black curve, tracing C).

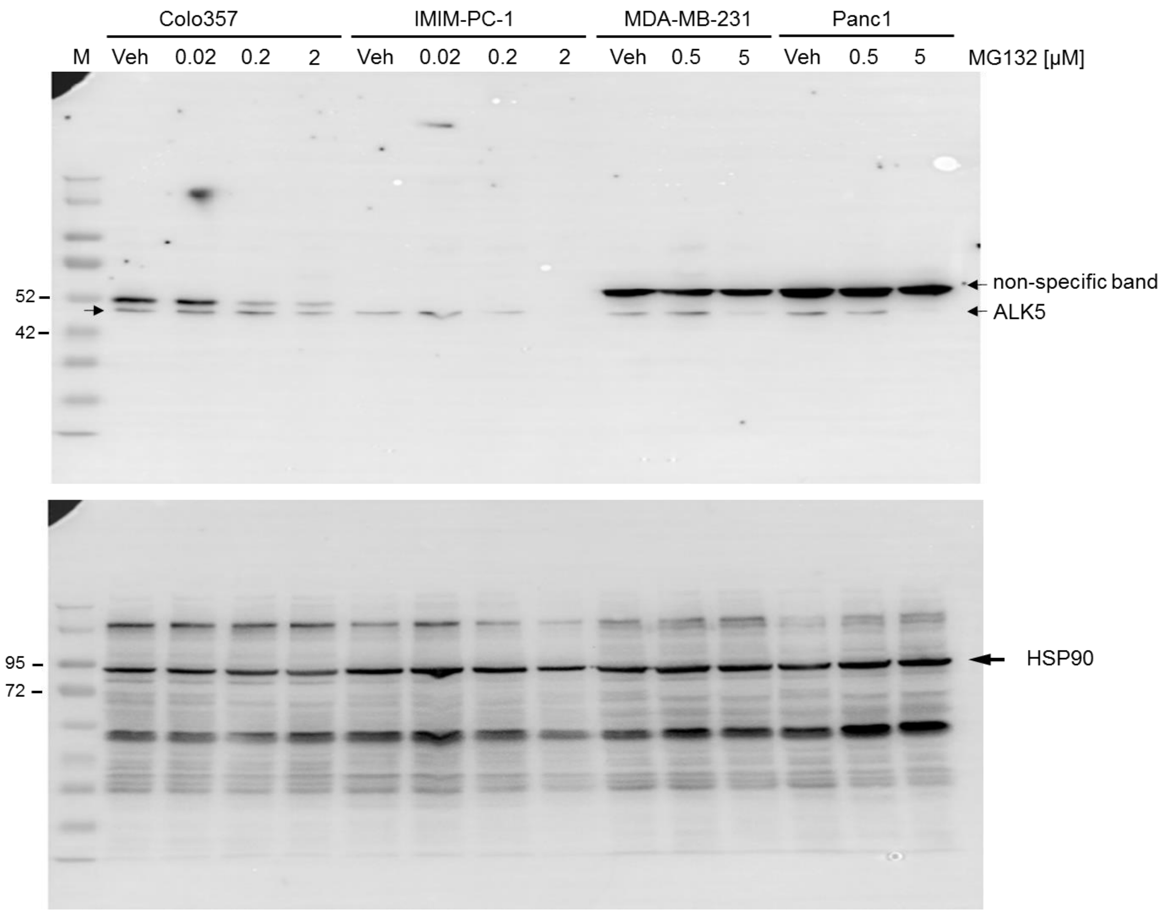

Figure S5. Uncropped blots of Figure 1B.

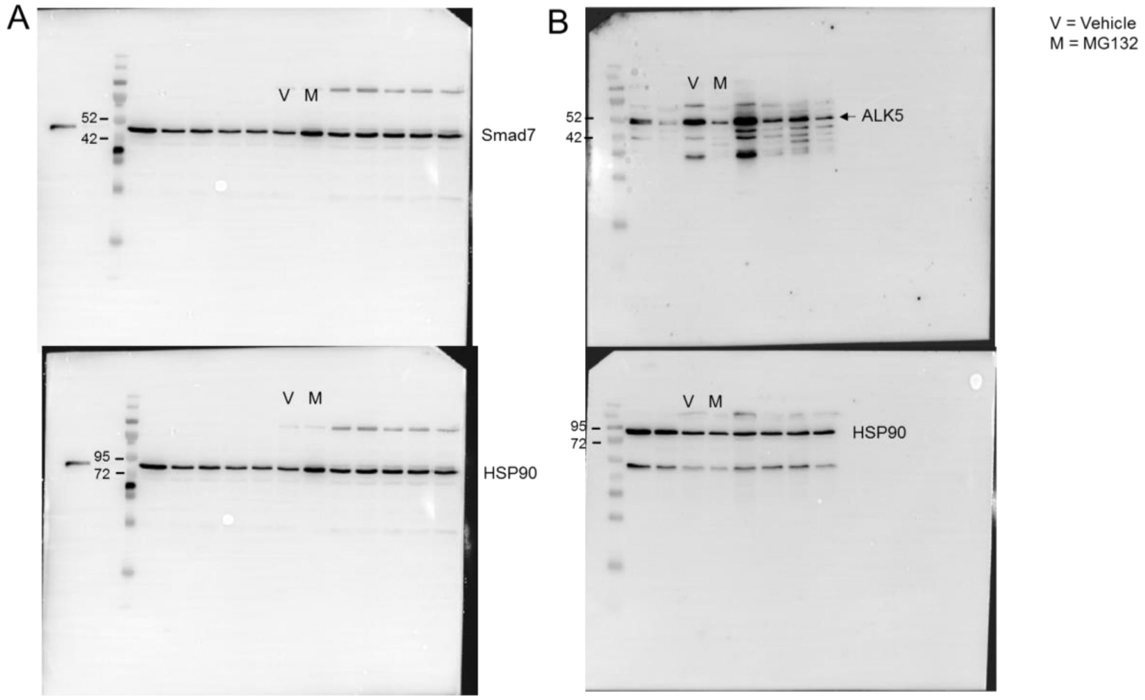

Figure S6. Uncropped blots of Figure 2.

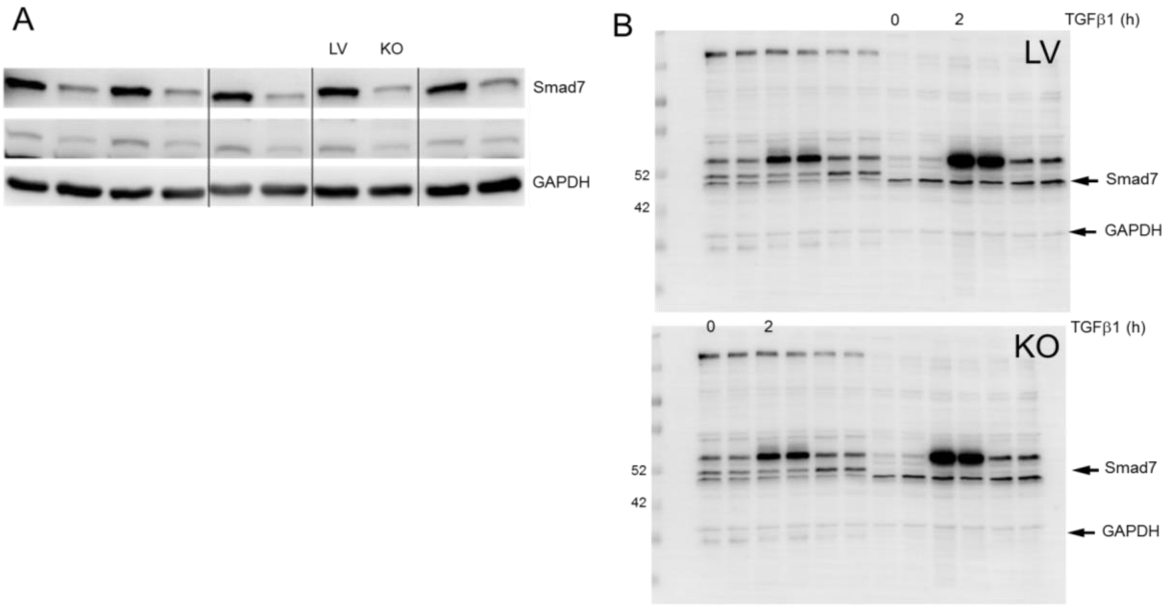

Figure S7. Uncropped blots of Figure 3.

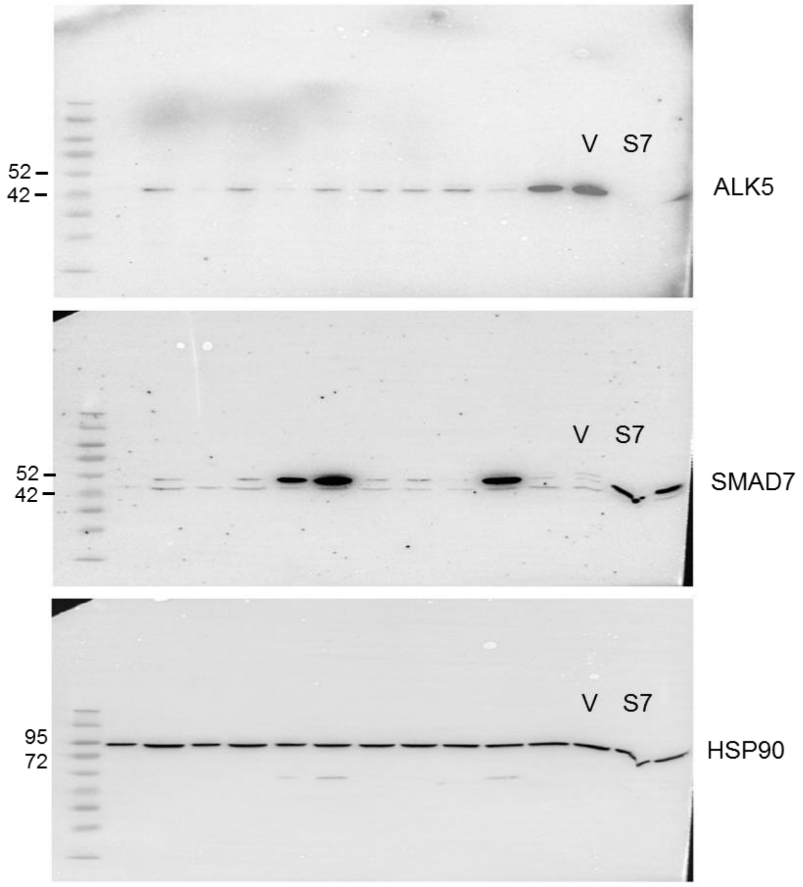

Figure S8. Uncropped blots of Figure 4.

Table S1. Primers used for qPCR.

Designation	Sequence (5′→3′)
ALK5-sense	GCGACGGCGTTACAGTGTTTCTGC
ALK5-antisense	ATGGTGAATGACAGTGCGGTTGTGG
MMP2-sense	CACCCTGGAGCGAGGGTAC
MMP2-antisense	CTGATTAGCTGTAGAGCTGAAGGC
USP4-sense	TTTCCTGGCCCAATAGACAAC
USP4-antisense	GGTAGGGACCAATACATAGTCCA
USP11-sense	AATCGTCAATGCCAGTGTACTT
USP11-antisense	TCTCATCGCAGTCAGGATCATAA
USP15-sense	CATTGAACGCAAGGTCATAGAGC
USP15-antisense	AACAGTGTGAGATTTGCCCAA
USP26-sense	TTGACACTTACTTGCGGAGAGT
USP26-antisense	ACACCTCGAACAATCTGCCTT
GAPDH-sense	TTGCCATCAATGACCCCTTCA
GAPDH-antisense	CGCCCCACTTGATTTTGGA
RAC1 exon 3b-antisense	GGCAATCGGCTTGTCTTTGCCC
RAC1-sense	ACCATGCAGGCCATCAAGTGTGTGG
SLUG-sense	ATATTCGGACCCACACATTACCT
SLUG-antisense	GCAAATGCTCTGTTGCAGTGA
SNAIL-sense	CTGCTCCACAAGCACCAAGAGTC
SNAIL-antisense	CCAGCTGCCCTCCCTCCAC
TBP-sense	GCTGGCCCATAGTGATCTTT
TBP-antisense	CTTCACACGCCAAGAAACAG
Vimentin-sense	TGGCACGTCTTGACCTTGAA
Vimentin-antisense	GGTCATCGTGATGCTGAGAA

© 2020 by the authors. Licensee MDPI, Basel, Switzerland. This article is an open access article distributed under the terms and conditions of the Creative Commons Attribution (CC BY) license (http://creativecommons.org/licenses/by/4.0/).
